# Dentition and body condition: tooth wear as a correlate of weight loss in roe deer

**DOI:** 10.1186/s12983-021-00433-w

**Published:** 2021-09-20

**Authors:** Roberta Chirichella, Anna Maria De Marinis, Boštjan Pokorny, Marco Apollonio

**Affiliations:** 1 Department of Veterinary Medicine, University of Sassari, Via Vienna 2, 07100 Sassari, Italy; 2 Italian Institute for Environmental Protection and Research (ISPRA), Via Ca’ Fornacetta 9, 40064 Ozzano dell’Emilia (BO), Italy; 3 Environmental Protection College, Trg mladosti 7, 3320 Velenje, Slovenia; 4 Slovenian Forestry Institute, Večna pot 2, 1000 Ljubljana, Slovenia

**Keywords:** *Capreolus capreolus*, Body mass, Cheek teeth, First molar height, Hypsodonty index, Scoring scheme, Tooth wear score, Senescence

## Abstract

**Background:**

In many mammalian species, once the permanent teeth have erupted, the only change to dentition is a gradual loss of tooth surface/height through wear. The crown of the teeth cannot be repaired once worn. When dental crown tissue has been depleted due to wear, the animal is expected to have a suboptimal body condition. We evaluated the role of tooth wear in causing a reduction of physical condition in adult roe deer females (*Capreolus capreolus*).

**Results:**

The progressive wearing of the lower cheek teeth was assessed in a Northern Apennines (Italy) population with a new scoring scheme based on objectively described tooth characteristics (morphotypes) being either present or absent. Eviscerated body mass and mandible length, which is a good proxy for body size in roe deer, were related to the tooth wear score by the use of linear regressions. The sum of wear scores for molariform teeth correlated most strongly with body condition (i.e., eviscerated body mass/mandible length), showing the importance of the entire chewing surface for acquiring energy by food comminution, chewing, and digestion. In comparison with individuals of comparable size experiencing minor tooth wear, the body mass of those with the most advanced stage of tooth wear was decreased by 33.7%. This method was compared to the height and the hypsodonty index of the first molar, the most commonly used indices of tooth wear. The sum of molariform wear scoring scheme resulted in a more suitable index to describe the variation in body condition of roe deer.

**Conclusions:**

Describing tooth wear patterns in hunted populations and monitoring at which tooth wear level (and therefore dental morphotype) an animal is no longer able to sustain its physical condition (i.e. when it begins to lose body mass) can be a useful tool for improving the management of the most widespread and abundant deer species in Europe. At the same time, such an approach can clarify the role of tooth wear as a proximate cause of senescence in ungulates.

**Supplementary Information:**

The online version contains supplementary material available at 10.1186/s12983-021-00433-w.

## Background

Senescence is defined as the gradual deterioration of function, accompanied by decreased survival and reproductive rates, with increasing age (e.g., [1, 2]). The disposable soma hypothesis was, among others, proposed to explain how senescence may evolve: it is based on optimal allocation of metabolic resources between somatic maintenance and reproduction [3]. Following this logic, all that is required is to keep the organism in sound condition for as long as it might survive in the wild [4, 5]. The necessary amount of energy needed for this purpose in wild animals is strongly linked to foraging and mastication efficiency that is, in turn, related to physical efficiency: efficient teeth are, in mammals, a prerequisite for regularly acquiring and processing food both in carnivores and herbivores [6].

Mastication is of paramount importance in the digestive processes of mammalian herbivores [7–9]. Reducing particle size through chewing increases the surface area on which symbiotic microorganisms can act, thereby increasing the fermentation rate and cell wall degradation [9, 10].

Permanent teeth in many mammalian herbivores do not grow once fully emerged, and their crowns cannot be repaired when damaged or worn [11, 12]. These types of teeth are used throughout an individual’s lifetime to comminute food up to the point when their crowns are fully eroded. Thus, at the time of eruption, teeth already have all their potential lifetime investment in durability. The compensatory mechanism of adding cementum at the roots of the teeth, as demonstrated in some ruminants [13, 14], might only serve to delay but cannot prevent the loss of dental functionality associated with wear.

In ungulates, tooth wear has been suggested to be a proximal cause of senescence because it can negatively affect body condition and individual performance [1, 2, 15–18]. Indeed, body condition and rates of survival and reproduction typically peak in prime-aged individuals in ungulates [19, 20]. All these traits of performance have been reported to decrease with advanced age (see [19–21] for survival; [22] for reproduction; [23, 24] for body condition). However, only a few studies have tried to quantify the pattern of wear for the molariform teeth using objectively described characteristics and, as far as we know, the relationship between wear in molariform teeth and body condition has not been investigated. According to the available literature, tooth wear was compared either between captive and free-ranging ungulates [25], among populations living in different climatic/environmental conditions [26–28] or having different diets [26, 27], showing more or less marked differences. The height of the first molar (M_1_) and the hypsodonty index applied to molars have been the most widely-used indices of tooth wear [1, 16, 29–31]. Subsequently, a description of the macroscopic effect of tooth wear by means of the mesowear method was introduced by [32] and [33], and has been applied to a very large number of extant and fossil species to infer a herbivorous diet. This method is based on the macroscopic observation of molar cusps that are worn by attrition, which is produced by one tooth occluding with its opposing tooth, and abrasion, which is caused by grinding food between teeth [34]. Recently, the mesowear method has undergone multiple applications, adaptations, simplifications, and extensions since its establishment [35]. However, in wildlife contexts this method cannot be experimentally applied as in domestic contexts [36–38].

Our study focuses on roe deer (*Capreolus capreolus*), the most widespread and abundant deer species in Europe, and a highly valued game species for which monitoring of population status is often required [39]. It is a relatively small ruminant [40] and, with its viscous saliva and unstratified rumen content, is generally classified as a strict browser along the browser-grazer continuum [41, 42]. However, its diet frequently contains ≥ 25% grass [43]. Considering that the wear pattern may vary according to the species, a scoring scheme was specifically developed for roe deer [44]. However, the wear process has not been studied in detail because ageing was the main aim of this research.

Our study aimed to: (1) propose a new tooth wear scoring scheme, describing the progressive wear of the cheek teeth based on objectively described characteristics being either present or absent; (2) determine if there is any evidence for a relationship between adult female body condition and the degree of tooth wear; (3) thus provide evidence of the role of tooth wear as a proximate cause of senescence determined as body weight loss in relation to animal (skeletal) size and select the best proxy of senescence using either tooth wear scores or the most widely-used indices of wear (i.e., the height of the first lower molar and the hypsodonty index).

In this context, describing the tooth wear pattern within a single population (i.e., a wild population with an uniform management regime and experiencing similar climatic/environmental conditions) and checking at which tooth wear level an animal is no longer able to sustain its optimal physical condition (i.e., when it begins to lose body mass till to reach values below the population average) can also facilitate comparisons of the life-history parameters of different populations that may live under varying environmental conditions (see [45]), and to understand the differential trends among them. This is especially important for income breeders such as roe deer [46], where females rely on food intake rather than fat reserves for reproduction [47]. Indeed, the income breeding strategy and the high level of maternal care in roe deer (e.g., [46]) increase the importance of being efficient enough to acquire energy throughout food comminution, chewing, and digestion not only for the survival of an individual mother but also for the long-term existence of viable populations.

## Methods

### Study area

**Fig. 1:**
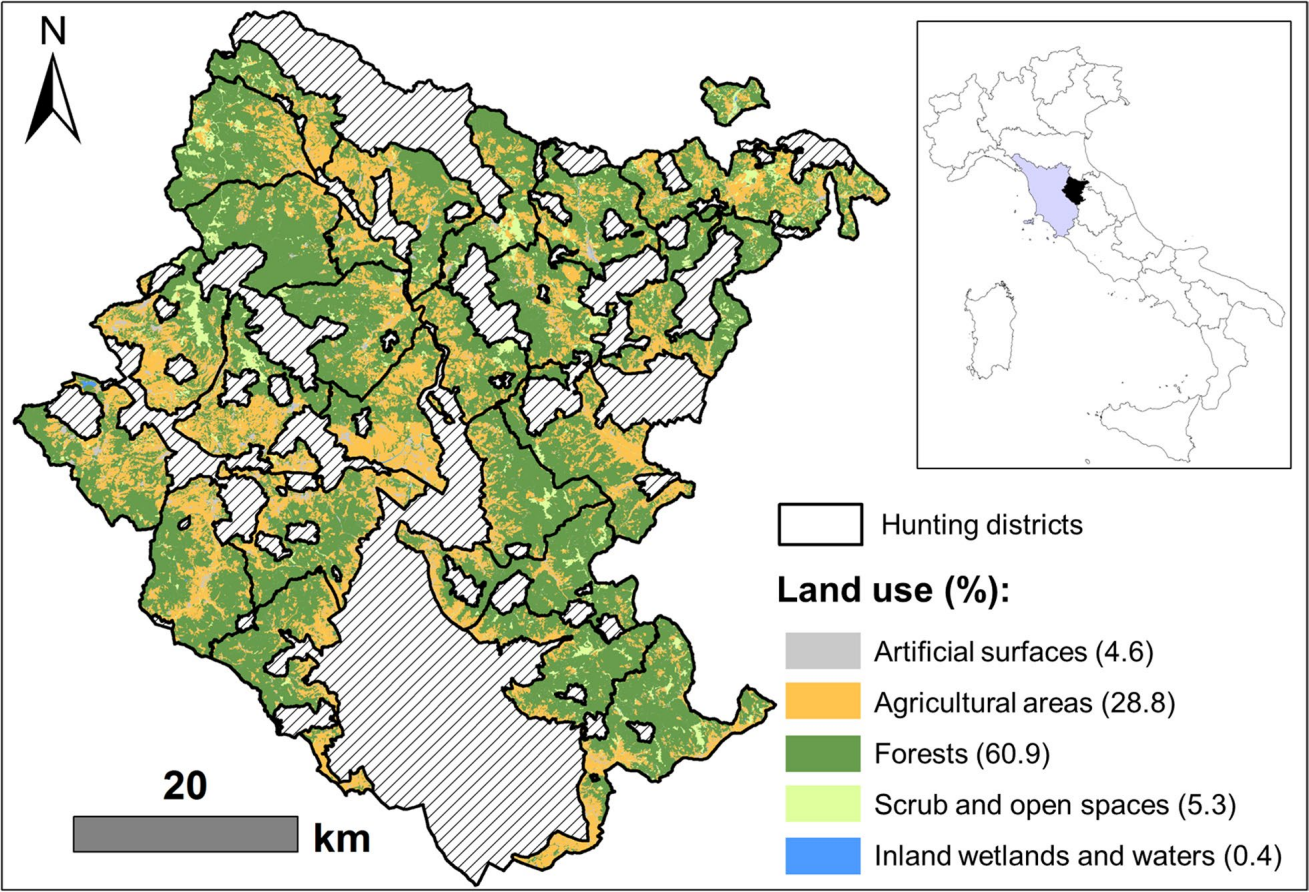
Study area. Map of the study site located in the Arezzo province (43° 28′ N, 11° 53′ E; black in the right panel), Tuscany (grey in the right panel), Central Italy. Study area includes 22 hunting districts (here classified into five land use classes according to Corine Land Cover 2012, left panel) where 319 roe deer females were legally shot during the regular annual harvest (1 Jan–15 Mar for females) between 2007 and 2017. Percentage of each land use class is reported

The study was carried out in the Arezzo province (Tuscany, Central Italy, 43° 28′ N, 11° 53′ E; Fig. 1). Approximately 57% of the territory is over 400 m above sea level (a.s.l.), with 7.4% of it being over 1000 m a.s.l. The climate is temperate continental, with a mean temperature ranging from 1.4 °C in January to 24.9 °C in July.

Roe deer hunting is only allowed in hunting districts: in the Arezzo province, there are 22 hunting districts of about 9500 ha each, subdivided into hunting zones of 109.35 ha ± 1.22 SD. The hunting districts are evenly distributed throughout the province and cover an overall area of 114,000 ha. The majority (61%) of this area is forested, predominantly composed of deciduous oaks (*Quercus cerris* and *Q. pubescens*) along with beech (*Fagus sylvatica*) and sweet chestnut (*Castanea sativa*). The remaining area consists of cultivated fields (29%) and scrub or herbaceous vegetation (5%) (see Fig. 1).

The study area harbors a rich wild ungulate community: besides roe deer, which is present in all the hunting districts, there are wild boar (*Sus scrofa*), fallow deer (*Dama dama*), red deer (*Cervus elaphus*), and mouflon (*Ovis gmelini musimon*). Wild boar is homogeneously distributed across the whole province, whereas red deer, fallow deer and mouflon are more localized [48]. The main predators are wolf (*Canis lupus*), with an estimated 25 packs, and red fox (*Vulpes vulpes*) [49, 50].

### Data collection

In all 22 hunting districts of the Arezzo province, roe deer females are legally hunted each year from January 1^st^ to March 15^th^. Hunters prepared all the mandibles of hunted individuals through hot water maceration method, hand removal of soft tissue and cartilage, and 35% hydrogen peroxide treatment. Each mandible was registered by the Provincial Government and made available to us for age assessment made by macroscopic inspection of teeth development and tooth wear [51], measurements and analyses. Age was determined using a tooth wear table developed locally, and validated age by histological examination of teeth through counting annual cementum layers in a sample set of > 300 individuals from a previous data collection [52]. However, because this method provides uncertain assessment of age of adult roe deer [53], we grouped animals into the age groups that have been commonly used in previous roe deer studies (e.g. [54, 55]): fawns and yearlings/subadults (< 20–22 months old; % available mandibles: 38%), 2-year-olds (32–34 months old; 25%), young adults (3–4 year; 25%), middle-aged adults (5–7 year; 8%), and old adults (≥ 8 year; 3%) (for major details about age estimation, see [55]). After regular annual evaluation, mandibles were stored according to the age class to which they belong and a sample of 319 roe deer mandibles was collected from adult females (i.e., older than 2 years) within the hunting season between 2007 and 2017 following the percentage of available mandibles in each age class. Only intact mandibles with intact teeth (i.e., not broken or chipped) were considered. Date of culling, sex, body mass (weight; BW), and hunting district were recorded for each roe deer. BW used in this study is eviscerated body mass (i.e., weighed without viscera and flowing blood). For the Arezzo province population, the mean eviscerated body mass of adult females culled in the Jan–Mar period was 18.1 kg [55].

In the laboratory, the length of the mandible (ML ± 0.01 mm) was measured as a proxy of skeletal size with a digital caliper. ML was measured (always by the same researcher) from the anterior margin of the alveolus of the first incisor (I_1_) to the posterior margin of the *Processus angularis* (see [56]). For the Arezzo province population, the mean value of ML for adult females was 156.7 mm (see [56]).

The progressive wearing of the cheek teeth was assessed using a scoring scheme based on objectively described morphotypes being either present or absent. The scoring scheme for roe deer that we proposed and used is described in Table 1. The proposed scoring scheme has been developed by AMDM and is based on 28 morphotypes recorded on two premolars (P_3_ and P_4_) and all three molars (M_1_, M_2_ and M_3_). P_2_ is the smallest premolar and has a simple crown in respect to other teeth that is only slowly changed by wear process [57, 58]. Therefore, this tooth was not included in the scoring scheme as it does not provide any additional value to the description of the cheek teeth wear process. The wear process that affects each tooth of the molar arcade has been schematized in stages representative of the entire process and identifiable by dental morphotypes whose characteristics are easily recognizable [59]. The schematization was based on the shape of the occlusal surface. When molariform teeth are unworn, this surface is characterized by three valleys in premolars and cusps separated by deep infundibula in molars (Table 1; see [12, 25, 60–63] for nomenclature and terminology of morphotypes of the occlusal surface). The progressive wear of the crown determines the reduction in the number of valleys and the gradual fusion of the cusps and the disappearance of the infundibula (Table 1; see [12, 25, 60–63]). Taking this into account, the wear process has been outlined in stages, each of which is identified by a recognizable morphotype depending on the number of valleys for the premolars and the fusion of cusps for molars (see Table 1 for major details about the recognizable morphotypes). Morphotypes are, therefore, characters that can be uniquely identified by any observer.

**Table 1:** Tooth wear scoring system

	Initial wear stage		Symbol	Description	Number of dental morphotypes
*Part a: dental morphotype*					
P_3_				Anterior (1), posterior (2) and back (3) valley	7
P_4_				Anterior (1) and back (3) valley	5
M_1_				Dentine exposed in all cusps but all dentine fields isolated	11
M_3_ Additional distal element (ADE)				Dentine exposed in the additional distal element but all dentine fields isolated	5

Tooth	Score	Description of wear stage	Symbol
*Part b: wear scoring system*			
P_3_	0	3 valleys	
	1	2 valleys and trace of the third one	
	2	2 valleys and no traces of the third one	
	3	1 valley and traces of the others	
	4	1 valley, traces of another one and no traces of the third one	
	5	1 valley and no traces of the others	
	6	Traces of 1 o 2 valleys and no traces of the others	
	7	No traces of valleys	
P_4_	0	2 valleys	
	1	1 valley and traces of the other one	
	2	Traces of the 2 valleys	
	3	Traces of 1 valley and no traces of the other one	
	4	Traces of valleys	
	5	No traces of valleys	
M_1_ M_2_ M_3_	0	Dentine exposed connects the lingual and buccal slopes of the cusps but all dentine fields remain isolated	
	1	Dentine exposed connects the lingual-mesial and buccal-mesial and the lingual-distal and buccal-distal slopes of the mesial or distal cusp and one infundibulum is closed	
	2	Dentine exposed connects the lingual-mesial and buccal-mesial and the lingual-distal and buccal-distal slopes of the mesial and distal cusp and both infundibula are closed	
	3	1 infundibulum closed and reduced to less than half and the other still open	
	4	1 infundibulum closed and reduced to less than half and the other closed and isolated	
	5	1 infundibulum closed and reduced to less than half and the other closed and not isolated	
	6	2 infundibula closed, not isolated and reduced to less than half	
	7	1 infundibulum no longer recognizable and the other still open	
	8	1 infundibulum no longer recognizable and the other closed and isolated	
	9	1 infundibulum no longer recognizable and the other closed and not isolated	
	10	1 infundibulum no longer recognizable and the other closed and reduced to less than half	
	11	Both infundibola no longer recognizable	
M_3_ Additional distal element (ADE)	0	Not connected with other cusps	
	1	Dentine exposed connects respectively buccal or lingual sides of the additional distal element with the lingual-distal or buccal-distal slopes of the distal cusps	
	2	Dentine exposed connects buccal and lingual sides of the additional distal element with the lingual-distal and buccal-distal slopes of the distal cusps	
	3	Dentine exposed connects buccal and lingual sides of the additional distal element with the lingual-distal and buccal-distal slopes of the distal cusps with back fossa well visible	
	4	Dentine exposed connects buccal and lingual sides of the additional distal element with the lingual-distal and buccal-distal slopes of the distal cusps with traces of back fossa	
	5	Dentine exposed connects buccal and lingual sides of the additional distal element with the lingual-distal and buccal-distal slopes of the distal cusps with back fossa no longer recognizable	

*Surfaces of the teeth. Diagram showing the terminology used to designate the surfaces of the teeth toward and away from the mandibular symphysis (mesial and distal) and toward the lips and the tongue (labial and lingual)

**Part a:** Dental morphotype. Dental morphotypes are based on the shape of the occlusal surface changing in relation to the progressive wear patterns of each considered tooth. Morphotypes of the initial wear stage are described and represented through symbols. The number of dental morphotypes that schematize the entire wear process is reported for each molariform teeth. See Part b for a comprehensive description of each wear stage and score). See [12, 25, 60–63] for nomenclature and terminology of morphotypes of the occlusal surface

**Part b:** Wear scoring system. Scores assigned to the progressive wearing stages of the cheek teeth based on objectively described morphotypes being either present or absent in each tooth. The molariforms wear score is the sum of all individual scores (see [12, 25, 60–63] for nomenclature and terminology of the occlusal surface)

**Fig. 2:**
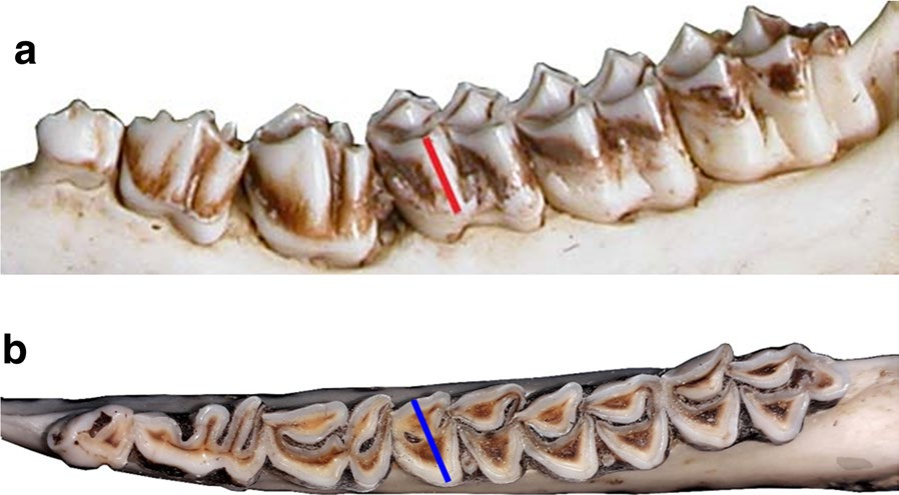
M_1_ height and width. **a** Height of M_1_ (red line) was measured with a digital caliper (HM_1_ ± 0.01 mm) as the distance from the peak of the mesiobuccal cusp to the enamel/cementum line (i.e., the stained part of the crown). **b** Width of M_1_ (blue line) was measured with a digital caliper (WM_1_ ± 0.01 mm) as the buccolingual breadth of the mesial cusp. Both HM_1_ and WM_1_ were measured 3 times per each female and mean values were derived ($\overline{{HM_{1} }}$ and $\overline{{WM_{1} }}$)

Tooth wear was also estimated using height of the first molar (M_1_) and the M_1_ hypsodonty index, which are the most widely-used indices of wear [1, 16, 30, 31]. In cervids, it has been noted that although crown formation in M_1_ is fully completed in the first months of life [64], the completion of eruption and final positioning of the molar in the mandible take place later [45, 64], and teeth also move in the mandible at very old age. Consequently, measuring molar height perpendicular from the mandible bone, labial or buccal side, is not necessarily a reliable measurement of molar wear [65]. Thus, the height (HM_1_ ± 0.01 mm) of M_1_ was measured with a digital caliper as the distance from the peak of the mesiobuccal cusp to the enamel/cementum line (i.e., the stained part of the crown; see Fig. 2). The width (WM_1_ ± 0.01 mm) of M_1_ was also measured with a digital caliper as the buccolingual breadth of the of the mesial cusp [66, 67]. Both HM_1_ and WM_1_ were measured 3 times per each female and mean values were derived ($\overline{{HM_{1} }}$ and $\overline{{WM_{1} }}$). Hypsodonty index (HI), defined as the height of M_1_ divided by the buccolingual breadth [66, 67], was calculated.

The dataset is available as Additional file 1.

### Data analyses

Eviscerated body mass and ratio of eviscerated body mass to total mandible length (i.e., BW/ML), as a proxy of body condition in roe deer [68–70], were related to the tooth wear score in 319 roe deer adult females, and compared to the mean value of eviscerated body mass and the body condition index for adult females of the same population [55, 56].

Linear regressions were calculated between BW and BW/ML (dependent variables) and (1) the wear scores of each tooth, (2) the sum of the scores obtained by pooling the molariform teeth in different ways (see Table 2 for the complete list of independent variables), (3) the height of M_1_ ($\overline{{{\text{HM}}_{1} }}$), and (4) the hypsodonty index of M_1_. Models were compared by means of Akaike’s Information Criterion for small sample sizes (AICc; [71]). Differences in body mass, mandible length and body condition index were investigated by ANOVA and Tukey HSD post hoc test for consecutive classes of tooth wear score (5 classes). The percentage of weight and body condition loss in relation to increasing tooth wear was calculated.

**Table 2:** Relation between female roe deer body condition and tooth wear indices

Independent variables	BW (kg)				BW/ML (kg/mm)			
	AICc (Rank, Value)	R^2^ adj	F	*p*	AICc (Rank, Value)	R^2^ adj	F	*p*
*Tooth wear scores*								
P_3_	7, 1329.0	0.221	91.26	< 0.01	7, -1838.6	0.211	86.28	< 0.01
P_4_	8, 1331.7	0.214	87.78	< 0.01	8, -1836.9	0.210	85.60	< 0.01
M_1_	9, 1334.1	0.209	84.80	< 0.01	9, -1830.1	0.197	78.98	< 0.01
M_2_	4, 1296.9	0.296	134.51	< 0.01	4, -1863.6	0.281	125.47	< 0.01
M_3_	5, 1298.5	0.292	132.22	< 0.01	5, -1859.9	0.268	117.41	< 0.01
M_3 (Additional Distal Element)_	10, 1354.7	0.156	59.65	< 0.01	10, -1821.0	0.169	65.60	< 0.01
M_3 total_	3, 1279.4	0.333	160.01	< 0.01	3, -1883.0	0.324	153.44	< 0.01
Premolars (P_3_ + P_4_)	6, 1307.4	0.272	119.89	< 0.01	6, -1858.5	0.263	114.57	< 0.01
Molars (M_1_ + M_2_ + M_3total_)	2, 1255.1	0.382	197.64	< 0.01	2, -1899.5	0.365	183.93	< 0.01
Molariforms (P_3_ + P_4_ + M_1_ + M_2_ + M_3total_)	1, 1247.3	0.397	210.48	< 0.01	1, -1907.6	0.381	196.48	< 0.01
*M*_*1*_* measurements*								
$\overline{HM1}$	11, 1359.5	0.145	55.13	< 0.01	11, -1806.2	0.122	44.98	< 0.01
Hypsodonty index (HI)	12, 1364.5	0.128	47.75	< 0.01	12, -1806.4	0.120	44.48	< 0.01

Main parameters of linear regressions between eviscerated body mass (BW in kg; on the left) and the ratio of eviscerated body mass to total mandible length (BW/ML in kg/mm; on the right) as a dependent variable and tooth wear scores, M_1_ height ($\overline{{{\text{HM}}_{1} }}$ in mm) and hypsodonty index of M_1_ (HI) for 319 roe deer females shot in the Arezzo province (Tuscany, Central Italy) during the regular annual harvest (1 Jan–15 Mar) between 2007 and 2017

Statistical analyses were performed in R version 4.0.4 (www.r-project.org, [72]).

## Results

**Fig. 3:**
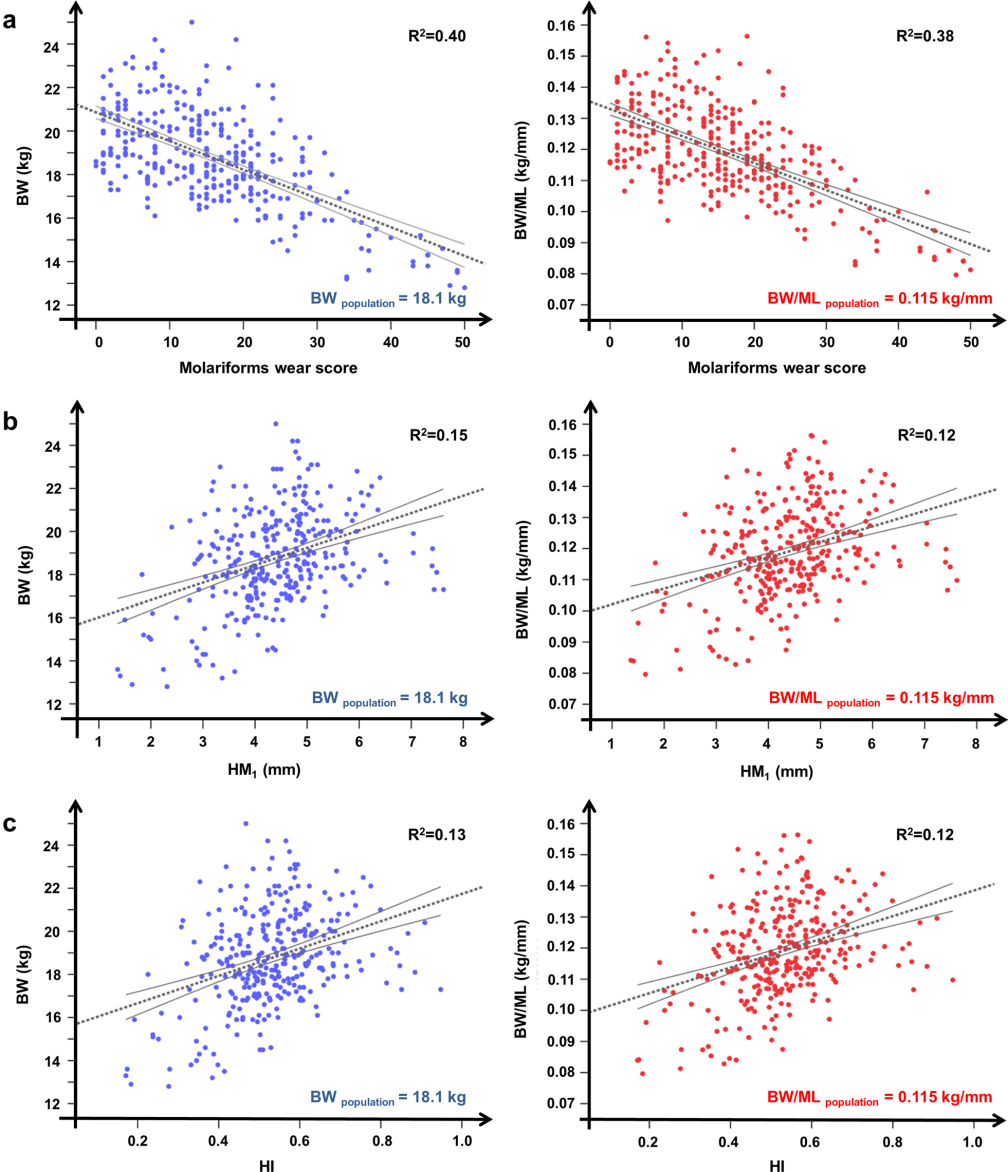
Female roe deer body condition versus tooth wear indices. Eviscerated body mass (BW in kg; on the left) and the ratio of eviscerated body mass to total mandible length (BW/ML in kg/mm; on the right) as a dependent variable in relation to: **a** molariforms wear score, **b** M_1_ height ($\overline{{{\text{HM}}_{1} }}$ in mm), and **c** hypsodonty index of M_1_ (HI of M_1_) for 319 roe deer females shot in the Arezzo province (Tuscany, Central Italy) during the regular annual harvest (1 Jan–15 Mar) between 2007 and 2017. R^2^ of the linear regression (dotted grey line) is reported

**Fig. 4:**
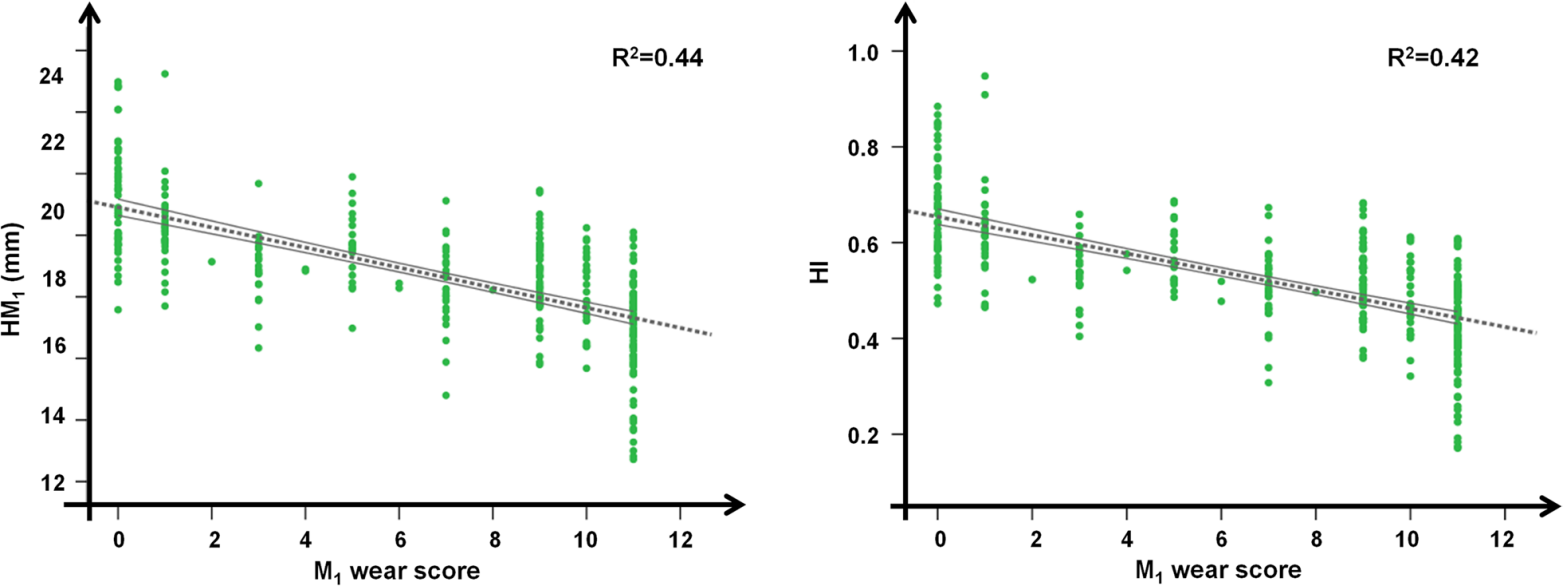
M_1_ height and M_1_ hypsodonty index versus M_1_ wear score. Height (HM_1_) and hypsodonty index (HI) of the first molar (M_1_) according to the increase in tooth wear score of M_1_ in 319 roe deer females shot in the Arezzo province (Tuscany, Central Italy) during the regular annual harvest (1 Jan–15 Mar) between 2007 and 2017. R^2^ of the linear regression (dotted grey line) is reported

Each evaluated proxy of weight and body condition loss showed a different ability to synthesize information for roe deer females (Table 2). Molars wear score (M_1_ + M_2_ + M_3total_) and molariforms wear score (P_3_ + P_4_ + M_1_ + M_2_ + M_3total_), which will be referred to in all subsequent parts of the paper, were the best correlated with body mass (Table 2, Fig. 3). On the contrary, and HI, despite their correlation with M_1_ wear score (*r*_*p*_ = − 0.67, *p* < 0.001 for $\overline{HM1}$ and *r*_*p*_ = − 0.65, *p* < 0.001 for HI; Fig. 4), were not suitable indices to describe the variation in body condition of roe deer (Table 2, Fig. 3).

**Fig. 5:**
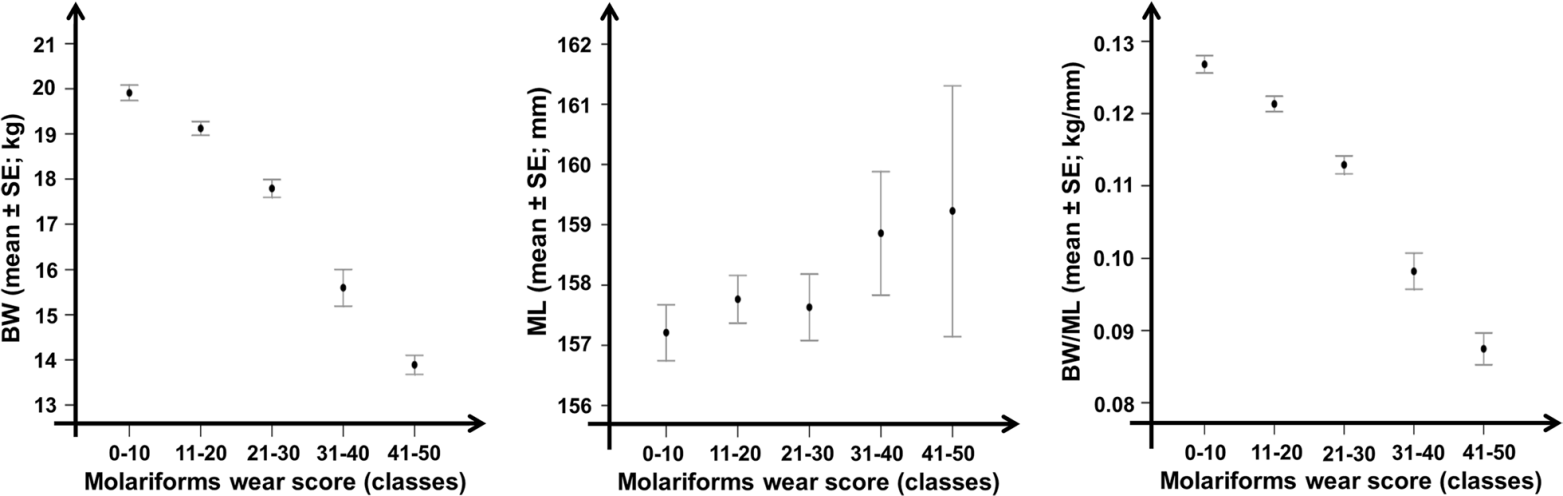
Female roe deer body condition versus tooth wear score classes. Eviscerated body mass (mean ± SE of BW in kg; left panel), mandible length (mean ± SE of ML in mm; middle panel) and the ratio of eviscerated body mass to total mandible length (mean ± SE of BW/ML in kg/mm; right panel) according to 5 tooth wear score classes for 319 roe deer females shot in the Arezzo province (Tuscany, Central Italy) during the regular annual harvest (1 Jan–15 Mar) between 2007 and 2017

**Fig. 6:**
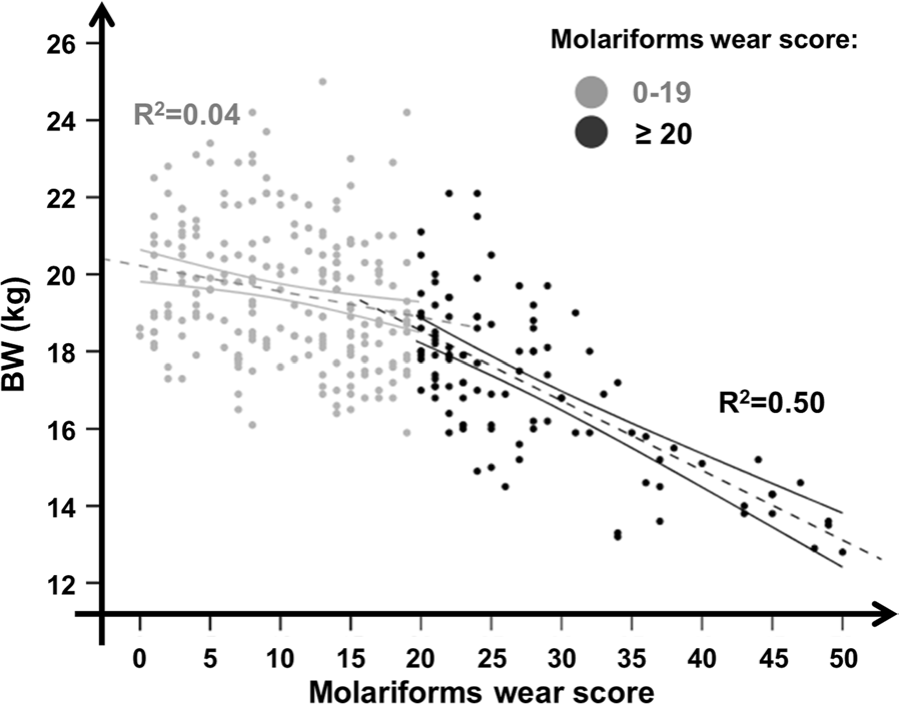
Eviscerated body mass versus molariforms wear score. Eviscerated body mass (BW) according to the molariforms wear score in 319 roe deer females shot in the Arezzo province (Tuscany, Central Italy) during the regular annual harvest (1 Jan–15 Mar) between 2007 and 2017. R^2^ of linear regressions are reported for females with a tooth wear score below (dot grey line) or above (dot black line) 20

Tooth wear score classes were related to significant loss of body mass and body condition (ANOVA: F_4,314_ = 56.85, *p* < 0.001 for BW; F_4,314_ = 47.57, *p* < 0.001 for BW/ML; for details, see Table 3 and Fig. 5), while no relation was found with mandible length (F_4,314_ = 0.85, *p* = 0.49; for details, see Table 3 and Fig. 5). Body mass and body condition decreased respectively by 33.7% and 30.8% in those with the most advanced stage of tooth wear compared to individuals experiencing minor tooth wear (Table 3). Eviscerated weight underwent a strong decrease when the molariforms wear score exceeded a value of 20. Individuals in the 21–30 wear score class had one or both infundibula closed in M_1_. With increasing wear score above this level, body masses were more and more frequently found to be below the population average (R^2^ = 0.50; see Fig. 6). This process was associated with the progressive disappearance of infundibula in M_2_ and M_3_.

**Table 3:** Eviscerated body mass, mandible length and a body condition index in different classes of the molariforms tooth wear score

	Tooth wear score classes				
				
	0–10 (N = 106)	11–20 (N = 119)	21–30 (N = 67)	31–40 (N = 16)	41–50 (N = 11)
*Eviscerated body mass (BW; kg)*					
Min	16.1	15.9	14.5	13.2	12.8
Med	19.9^a^	19.1^b^	17.8^c^	15.6^d^	13.2^e^
Max	24.2	25.0	22.1	19.0	15.2
% loss	–	4.0	10.6	21.6	33.7
*Mandible length (ML; mm)*					
Min	142.97	147.23	146.39	151.07	143.02
Med	157.21	157.77	157.63	158.86	159.23
Max	167.91	167.43	166.56	168.24	167.53
% loss	–	–	–	–	–
*Body Condition Index (BW/ML; kg/mm)*					
Min	0.10	0.10	0.09	0.08	0.08
Med	0.13^a^	0.12^b^	0.11^c^	0.10^d^	0.09^e^
Max	0.16	0.16	0.15	0.12	0.11
% loss	–	7.7	15.4	23.1	30.8

Minimal, average and maximal values of eviscerated body mass (BW; kg), mandible length (ML; mm) and a body condition index (BW/ML; kg/mm) of 319 roe deer females shot in the Arezzo province (Tuscany, Central Italy) during the regular annual harvest (1 Jan–15 Mar) between 2007–2017 according to different classes of the molariforms’ tooth wear score. Percentage of weight loss and body condition loss were calculated referring to individuals with minor tooth wear, i.e. those belonging to the score class 0–10, and according to the differences in consecutive tooth wear score classes (F_4,314_ = 56.85, *p* < 0.001 for BW; F_4,314_ = 47.57, *p* < 0.001 for BW/ML). Tukey HSD post hoc test results are indicated by superscripts (a-e)—means within a row not sharing the same superscript differ significantly. No differences in mandible length (F_4,314_ = 0.85, *p* = 0.492) for consecutive tooth wear score classes were revealed. Pictures show the mean wear condition of each tooth wear class

## Discussion

In roe deer, once the eruption process is completed, teeth undergo changes leading to a gradual loss of occlusal surface and a progressive decrease in crown height through a wearing process. Teeth cannot be repaired externally once worn [11, 12], despite a wear-compensation mechanism in dental roots confirmed in some ruminants (i.e., crown volume loss correlated to root volume gain; [13, 14]). These dental characteristics allowed us to test a new proposed tooth wear scoring scheme, describing the progressive wear of the cheek teeth.

The morphotypes described can represent the wear process, and the wear score classes identify stages that correspond to a progressive loss of dental functionality. This scoring system could give different results depending on the population because the turnover of morphotypes is obviously influenced by the characteristics of each individual as well as by the experiences and habits acquired in a specific type of environment [26–28]; with advancing age all this takes on greater importance, because the different ecological factors experienced by each individual accumulate over the years [59].

Our results clearly indicate that there is a relationship between adult female body condition and the degree of tooth wear. Moreover, the wear score of molariform teeth correlated the best with the body mass and body condition of adult females, showing the importance of the entire chewing surface in shaping the ability of the animal to acquire energy throughout food comminution, chewing, and digestion.

Many studies of wild ungulates have documented age-related changes in adult body mass which may decline through senescence of physiological function and reduced foraging ability (reviewed in [73, 74]) linked to advancing tooth wear (e.g., [2, 16]). Indeed, tooth wear is a proxy of food intake investment that has been confirmed, for example, in red deer. In this species, stags with more worn teeth generally grow larger antlers for their age [75] and individuals of both sexes with more worn teeth have a larger skeletal body size for their age [64, 76]. At the same time, because teeth in deer cannot be replaced or repaired during the animal’s life, tooth wear becomes a proxy for animal lifespan [2, 18, 64, 65, 75, 77]. Indeed, well-functioning molariform teeth are essential for digestive processes in ruminants, and their functionality and durability are of major importance to animal performance. This is the case for many deer species (see [2, 18, 73, 78, 79] for red deer; [67] for sika deer (*Cervus nippon*); [1] for moose *Alces alces*; [16, 31] for reindeer *Rangifer tarandus*; [17] for roe deer). For example, in Slovenia roe deer females with the most advanced tooth wear (recognized as elderly individuals) on average weighed 1.7 kg (10.5%) less than females with moderate tooth wear [54].

Besides a significant influence on body mass, particularly in individuals with the most worn teeth as shown by our results (see Figs. 3, 6), reduced foraging ability due to advanced tooth wear may indirectly (via body mass related effects) influence some other life-history traits such as reproductive ability. In female ungulates, reproductive performance depends on a broad set of extrinsic and intrinsic factors (e.g. [80]). One of the most important factors is body condition, which depends, among other things, on individual history (i.e. age, reproductive history, and fertility; e.g. [81, 82]) and environmental-climatic conditions (i.e. habitat quality, weather conditions, and population density; e.g. [83, 84]). Individual characteristics and food availability (quantity and quality) seem to be the most important factors because they shape how body reserves are invested, including in reproduction (e.g. [85, 86]). As a consequence, reproductive success in female ungulates is strongly related to body mass in many species, such as wild boar *Sus scrofa* [87], caribou/reindeer [88], moose [89], and red deer [90].

In the case of our studied species, the roe deer, a pronounced influence of female body mass on reproductive success was clearly confirmed both within several European populations (reviewed in [91]) and among populations across Europe [92]. For example, in a similar environment to our study, [54] demonstrated that ovulation in roe deer females increases with higher body mass, while for our study area (Tuscany, Italy), [55] verified the important role of body mass in implantation success. These findings strongly confirm the pronounced influence of female body mass on reproductive success not only considering their potential at a very initial phase of reproduction (i.e. ovulation), but also when considering reproductive allocation in later and more costly phases (i.e. implantation). Furthermore, the positive effect of body mass on female reproductive performance, including implantation success, weakens with age, and aging strongly influences the overall reproductive performance of roe deer females. Lower ovulation ability in very old does [54], increases in implantation failure [55, 93, 94], and overall decrease in fertility and smaller litters with aging [19, 95] indicate the existence of reproductive senescence in roe deer females. As all these parameters of reproductive success are strongly related to female body mass (reviewed in [92]), our results suggest that reproductive senescence could be related to the reduced efficiency of teeth due to their excessive wear.

Finally, our study allowed us to compare our new tooth wear scoring scheme with the other widely-used indices of wear (i.e., the height and the hypsodonty index of M_1_). Both measurements are confirmed as indices that are able to provide general information on the wear pattern. However, they fail to synthesize the wear stages of the entire occlusal surface of the molar arcade and less correlate the variation in body conditions. These findings could be related to the high degree of wear of the first molar in the last years of life for deer species that doesn’t allow much differentiation among individuals [58, 59].

Future work should aim to connect the true age of an individual with the tooth wear classes in order to verify when aging starts to be evident both through increases in tooth wear and decreases in body condition. This approach applied to populations of the same species living in different environments may help to elucidate intraspecies variability in population dynamics. Moreover, collecting longitudinal data with repeated tooth wear scoring in animals within the same population over longer timespan series (i.e., in different cohorts) may improve our ability to quantify the costs related to important life-history events such as reproduction, and to provide information about potential annual/periodic variation in tooth wear.

## Supplementary Information

**Additional file 1.** Dataset. Individual characteristics, tooth wear scores, M_1_ height and hypsodonty index of M1 data referred to 319 roe deer females legally shot during the regular annual harvest (1 Jan–15 Mar for females) between 2007 and 2017 in the Arezzo province (43° 28′ N, 11° 53′ E), Tuscany, Central Italy

## Data Availability

All data generated or analysed during this study are included in this published article and its additional files.
